# Phenomenological analysis on whipping behavior of rice flour batter

**DOI:** 10.1111/1750-3841.15452

**Published:** 2020-11-06

**Authors:** Chiaki Ichikawa, Daitaro Ishikawa, Jia Min Yang, Tomoyuki Fujii

**Affiliations:** ^1^ Graduate School of Agricultural Science Tohoku University Sendai Miyagi 980‐8572 Japan; ^2^ Faculty of Agriculture Fukushima University Fukushima Fukushima 960‐1296 Japan

**Keywords:** bubble size, dimensionless equation, dynamic wettability, three‐phase dispersion, whipping

## Abstract

**Abstract:**

In this study, the bubbles in rice flour batter were investigated under a constant temperature, because the bubble size distribution is important for the control of food texture. We obtained experimental data using a hand mixer and compared the properties of doughs prepared using six rice flours; each flour was prepared through a different milling process. We also added the size effect of the rice flour particles as the Bond number. Furthermore, we performed a dynamic wettability test to estimate the wettability of the rice flour surface. The results of this test were described well by the Washburn equation, and *d_c_cosθ/d_p_* was calculated as a wettability parameter (where, *d_c_* = effective diameter of a capillary in a powder bed, *cosθ* = the contact angle, *d_p_* = mean particle diameter of rice flour). If bubble sizes depend mainly on the inertial force, viscous force, surface tension, and gravity, then the normalized mean bubble diameter should be a function of the Reynolds number, Weber number, and Froude number. The mean bubble diameter (*d_bm_*) generated by whipping was expected to be affected by the thickness (*d*) of the rod of the mixer, its movement speed, and physical properties of the material. Therefore, dimensionless mean diameter (*d_bm_/d*) was expressed based on a dimensionless equation. In the three‐phase dispersion, different empirical equations were obtained depending on the amount of rice flour added, and the bubble diameter could be predicted using dimensionless parameters. In addition, the equations were generally applicable to the various materials selected for this study.

**Practical Application:**

The powder properties of rice flour were investigated, and dimensionless parameters were analyzed to construct an appropriate process control system for rice flour‐based food products. Although the process method optimized for flour products is also used for rice flour products in practical situations, the comprehensive evaluation based on dimensionless parameters leads to optimization of the process for rice‐flour based products. Moreover, this optimization might strongly support the creation of a new texture, and thus, the potential for market expansion of rice‐flour based products is considerable.

## 1 INTRODUCTION

Rice flour has many properties that are advantageous for human health, such as high digestibility, low calories (low absorption potential of oil), and functional components, when compared to wheat flour (Ohtsubo, 2014; Sanchez, Osella, & de la Torre, 2006). In particular, the increase in the incidence of celiac disease, which is a digestive issue caused by an immunological reaction to gluten, has increased the demand for gluten‐free products over the last two decades (Rai, Kaur, & Chopra, 2018). Rice flour is a key alternative to flour for minimizing food allergies, and many products based on rice flour, such as breads, cakes, and noodles, are commercially available. However, gluten is an important determinant of the texture of food products because its network structure holds air and provides elasticity.

The texture of rice flour‐based products without gluten is typically moist and chewy. Although this texture is unique and appealing, its inflexibility is an obstacle to the expansion of rice flour‐based products. Moreover, the processing of cellular foods is usually optimized for flour, and thus, favorable texture of rice flour‐based food products is not obtained by simply replacing flour with rice flour. Therefore, studies on the development of gluten‐free cellular foods are conducted by imitating the elastic properties of gluten with food additives such as emulsifiers, thickeners, and nonstarch polysaccharides (Chesterton, PereiradeAbreu, Moggridge, Sadd, & Wilson, 2013; Fitzgerald, Martin, Ward, Park, & Shead, 2003; Hedayati & Tehrani, 2018; Okunishi, 2015; Rodríguez‐García, Salvador, & Hernando, 2014; Sabanis & Tzia, 2011; Sahi & Alava,2003; Sivaramakrishnan, Senge, & Chattopadhyay, 2004; Torbica, Hadnađev, & Dapčević, 2010). While there is a thorough understanding of the chemical properties of air bubbles in foods, few studies have focused on their physical properties; hence, they have not yet been sufficiently analyzed (Campbell & Mougeot, 1999; Elgeti, Jekle, & Becker, 2015). In particular, the optimization of process control methods is essential for the development of new rice flour‐based foods with desirable textures.

Our research group has studied the powder properties of rice flour subjected to different milling techniques to appropriately control the production process. Fujii and Shoji (2012) reported that the surface and internal structure of rice flour subjected to milling form a core‐shell structure. Shoji et al. (2012) also found that the hydration behavior is closely related to the internal structure of rice flour. Moreover, Ishikawa et al. (2017) demonstrated that adsorption properties might affect the internal structure and showed the potential of developing optimally controlled rice flour production based on mechanical shock in the milling process. However, precise analysis of the process control methods for rice flour production is still needed.

Rice flour‐based cellular foods have been prepared from batter with air‐liquid‐solid phases. In particular, rice flour batter without gluten has a low viscosity and many bubbles are generated in the batter through the whipping operation. Therefore, investigations of the distribution of bubble sizes during the whipping process are important for optimizing the texture. Although the whipping operation has been widely applied to cooking and food processing, there is limited fundamental engineering knowledge that can be used as a basis for selecting operating conditions and designing equipment.

Understanding multidimensional phenomena using dimensionless parameters is a well‐known technique in chemical engineering. Yano (1987) predicted the mean foam bubble diameter generated using various whippers, from electric household whippers to intermediate industrial‐scale practical machines, as well as in several protein solutions and surfactant solutions (Yano, 1987, 1988). The mean bubble diameter generated by whipping is considered to be affected by multiple factors such as the physical properties of the sample and the mechanical and operational conditions during agitation. It is expected that the equation can be applied to versatile situations using different materials, concentrations, and whippers, allowing for the engineering prediction of whipping. However, this has not been fully studied for three‐phase dispersions containing solids such as the batter of cellular foods. Therefore, the aim of this study was to evaluate the whipping properties of three‐phase batter containing rice flour and soymilk. In this study, the powder properties of rice flour were investigated, and dimensionless parameters were analyzed to construct an appropriate process control system for rice flour‐based food products.

## 2 MATERIALS AND METHODS

### 2.1 Materials

White rice (cv. Hitomebore) cultivated in 2015 at Miyagi Perf., Japan was used as the material of rice flour. Six rice flours, referred to as RFa–RFf, were prepared. RFa was produced by initial milling to 3.0 mm or less with a screen and secondary milling at 0.3 mm under 3,600 rpm using a pin mill device (Pulverizer Makino‐type DD‐2, Makino Mfg. Co., Ltd., Tokyo, Japan). RFb was pulverized by high‐speed milling at 10,000 rpm using an exceed mill device (Exceed mill EM‐2, Makino Mfg. Co., Ltd.). For RFc, the white rice was milled by pin milling at 3,600 rpm and then pulverized by high‐speed milling at 11,000 rpm by the wet grinding method with 5% water by weight of rice flour. RFd was produced by pin milling at 3,600 rpm and then by high‐speed milling at 10,000 rpm. RFe and RFf were pulverized under the same conditions, except the milling time. The milling time for RFe was twice as long as that for RFd; for RFf, it was four times longer than that for RFd. Rice flours, excluding RFc, were prepared by the dry grinding method. To investigate the effects of the liquid phase, sucrose was added to unadjusted soymilk containing 12% w/w soy solids (Kitano Daizu, Taishi Food Inc., Aomori, Japan), and 10 to 40% w/w solutions were prepared.

### 2.2 Methods

#### 2.2.1 Powder properties

The particle size distribution was measured using a particle size analyzer (Laser Micron Sizer LMS‐2000e; SEISHIN Enterprise Co., Ltd., Tokyo, Japan) by the laser diffraction method. This instrument has a helium neon laser and a solid laser as red and blue light sources. Consequently, particle size can be measured at the submicron order.

The particle density of each powder sample was measured by the liquid displacement method using a Gay‐Lussac‐type Pycnometer (Model 82–2355; Sansyo Co., Ltd., Tokyo, Japan). 2‐Propanol was used as the dispersion medium, and vacuum drying was performed at 0.07 MPa for 5 hr. Density was measured at 25 °C, and the specific weight of 2‐propanol was 0.78084 g/cm³.

The dynamic wetting test of each powder sample was performed using a hydration meter (type: TU‐01; Hatsuratsu Co., Sendai, Japan). A sample cup 40 mm in diameter and a height of 30 mm was filled with the sample powder and set on a transparent tank. The tank was connected to a water bath. The water surface height in the bath was reduced by hydration toward the powder bed by capillary pressure. The water was supplied from a reservoir because the water level returned. The time course of the volume of water supplied was monitored for 3 hr at intervals of 15 s with a cumulative flow meter. The course of the squared hydrate volume was fitted by the Washburn equation (Eq. 1), which explains capillary penetration. In this study, hydration of the powder bed was regarded as a capillary phenomenon (Holmberg, Shah, & Schwuger, 2006).

(1) $$V\;=\sqrt{{\left(S\cdot \varepsilon \right)}^{2}\frac{r\cdot \gamma_{w}\cos\theta }{2\cdot \eta_{w}}\;t}\;\;$$

where *V* is the hydration volume, *t* is the hydration time, *S* is the cross‐sectional area of the powder bed, *ε* is porosity, *r* is the capillary radius, *γ_w_* is the water surface tension, *η_w_* is the water viscosity, and *θ* is the contact angle. In this study, *γ_w_* and *η_w_* were set to 72.5 mN/m^2^ and 1.0026 mPa·s, respectively.

The slope of the hydration curve obtained by the change in *V* over time was used to obtain the wetting parameter *Ccosθ*, as follows:

(2) $$Ccos\theta =\left(Slope\right)\cdot \frac{\eta_{w}}{\gamma_{w}}$$

Simultaneously,

(3) $$\;\;C\;={\left(S\cdot \varepsilon \right)}^{2}\;\cdot \frac{r}{2}$$

Therefore, when the capillary radius (*r*) and porosity (*ε*) are constant during the hydration period, the wettability parameter *d_c_cosθ* can be quantitatively defined. In this study, the capillary diameter (2*r*) was defined as *d_c_*.

#### 2.2.2 Physical properties of the liquid phase

To prepare solutions of different surface tensions and viscosities, sucrose in the concentration range of 0 to 40% was added to soymilk and agitated using a magnetic stirrer. Moreover, sodium dodecyl sulfate (SDS) aqueous solution was prepared, so that its concentration became 0.3% w/w, and it was selected as a model foam. Sucrose in the concentration range of 40 to 60% was added to the SDS solution.

The surface tension of the solution was measured using a surface tension meter (Surface Tensiometer LBVP‐A3; Kyowa Kaimenkagaku Co., Ltd., Tokyo, Japan). The viscosity was measured using a Brookfield viscometer (Tokyokeiki, Tokyo, Japan), and fluidity curve measurements were obtained at a rotational speed of 6, 12, 30, and 60 rpm. The viscosity calculated from the value at 60 rpm was regarded as the viscosity of each solution. The density was measured following the methods described in Section 2.2.1.

#### 2.2.3 Whipping properties of rice flour‐based batter prepared with soymilk

Both solutions of soymilk or SDS with sucrose were subjected to mixing using a hand mixer (Tescom THM26) at 800 to 1,250 rpm for 1 min, and then, 5 to 50 g of each rice flour was added to the solution. Each solution was subjected to mixing using the same whipper at the same rotational speed as the initial whipping rotaion speed (800 to 1,250 rpm) for 10 min, and rice flour‐based dough with soymilk or SDS was prepared. The bubbles in the rice flour batter were investigated under a constant temperature. The sample was dropped on a slide glass with a spatula immediately after preparing the batter, covered with a cover glass, and settled on a microscope equipped with a CCD camera. Images of 1,740 × 1,180 pixels were obtained and bubbles were identified in each image by tracing the contours manually. The images were transferred to analysis software, Gel‐Pro® Analyzer (Media Cybernetics, Inc., Silver Spring, MD, USA), and the diameter of bubbles was measured. A total of 512 bubbles were measured in each sample, and the mean bubble size was calculated.

## 3 RESULTS AND DISCUSSION

### 3.1 Evaluation of powder properties by dimensionless parameters

Particle size measurements for rice flours are shown in Table 1. The volume‐based mean particle sizes ranged from 52.73 to 241.9 µm. We calculated number‐based mean particle size (*d_p_*) from the volume‐based particle size distribution of each sample as follows:

**Table 1:** Summary of volume‐ and number‐based mean particle sizes for each rice flour. (Unit : µm)

	D[4,3]	d(0.1)	d(0.5)	d(0.9)	*d_p_*
RFa	185.2	49.83	176.4	325.1	6.076
RFb	241.9	43.97	165.6	493.8	7.218
RFc	198	34.36	152.4	376.4	5.631
RFd	52.73	9.632	44.25	108.2	1.65
RFe	144	19.46	86.9	225.6	2.158
RFf	226.1	31.55	134.3	480.7	5.442

D[4.3], volume‐based mean particle size; d(0.1), maximum particle diameter is less than 10% of the sample falls; d(0.5), maximum particle diameter below which 50% of the sample falls; d(0.9), maximum particle diameter below which 90% of the sample falls; *dp*, number‐based mean particle size calculated from D[4,3].

At first, the number‐based frequency (*r_j_*) was decided from the measurement results.

$$\;r_{j}=\frac{p_{j}}{s}\;\times \;100\;\;p_{j}=\frac{q_{j}}{\frac{4}{3}\pi {\left(\frac{zj}{2}\right)}^{3}}\;\;s\;=\sum_{j=1}^{h}p_{j}\;$$

where *q_j_* is the volume‐based frequency, *z_j_* is mean particle size for each section, and *h* is the number of particle size divisions. Then, the number‐based mean particle size was calculated with the distribution.

$$\;d_{p}=\frac{\sum_{j=1}^{h}z_{j}\times r_{j}}{100}\;$$

The number‐based mean particle size was 1.650 to 7.218 µm. In this study, we used the number‐based mean particle size as the mean particle size (*d_p_*) of each sample. The surface state of a particle is described by the Bond number (*Bo*). To investigate the surface state of rice flour, *Bo* based on a particle was calculated by the ratio of the contribution of gravity to the surface tension, as follows:

(4) $$Bo\;=\frac{\rho_{p}\;d_{p}^{2}\;g}{\gamma_{L}}\;$$

where *Bo* is the Bond number based on a particle, *ρ_p_* is the particle density, *d_p_* is the particle diameter, *g* is gravity, and *γ_L_* is the liquid surface tension. *Bo* was calculated using the density of particles, surface tension of water (0.0725 N/m), and gravity (9.81 m/s²), and it was in the range of 6.2 × 10^−7^ to 1.2 × 10^−5^.

**Figure 1:**
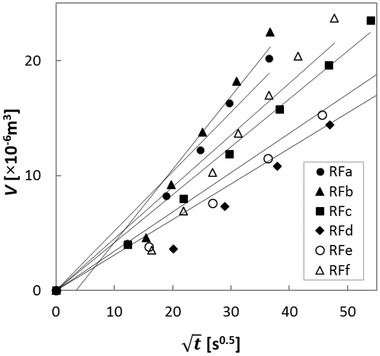
Course of the volume of hydration over time.

**Figure 2:**
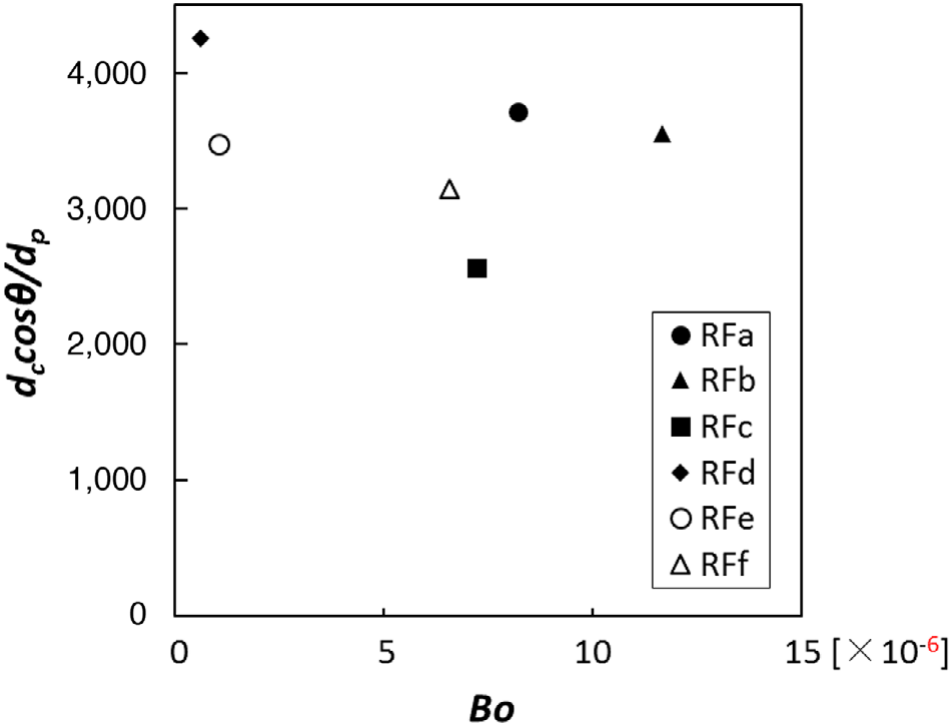
Plot of *Bo* and dimensionless parameter for wettablilty *d_c_cosθ/d_p_*.

The course of the squared hydrate volume is summarized in Figure 1. A linear increase in the squared volume of liquid was observed. This trend was well described by the Washburn equation (see Eq. 1). The wettability parameter *d_c_cosθ* was calculated using the slope of regression in Figure 1. It was assumed that *d_c_cosθ* is affected by particle size; thus, a dimensionless wettability parameter was obtained via the particle size (*d_p_*). The dimensionless parameter of wettability *d_c_cosθ/d_p_* ranged from 2,561 to 4,260 among rice flours. A two‐dimensional plot of the *Bo* and *d_c_cosθ/d_p_* is shown in Figure 2. The points were clearly distributed in the two‐dimensional map showing *Bo* and *d_c_cosθ/d_p_*. Thus, these results suggested that rice flour subjected to different milling procedures could be classified by its *Bo* and its *d_c_cosθ/d_p_* value.

### 3.2 Physical properties of the liquid phase

A typical cake batter is composed of flour, eggs, sugar, fats and oils, an emulsifier, and water as the main materials. When this batter is observed with a microscope, it can be confirmed that the egg aqueous solution containing a high concentration of sugar exists as a continuous phase and that many bubbles spread as a dispersed phase. Ochi and Yoshikawa (1969) evaluated the effect of the compounding ratio on the quality of sponge cakes and concluded that sugar, eggs, water, and flour are the most influential raw materials. Since sugar is dissolved in eggs and water, the physical property of the liquid phase, which is the continuous phase of the batter, is one of the factors that greatly affect the quality of the final product. The bubble is stabilized by appropriate viscosity of the liquid phase when subjected to stirring, while the bubbles disappeared with high viscosity (Fujii & Danno, 1988). Therefore, the surface tension, viscosity, and density of solutions were measured as the physical properties of the liquid phase.

The surface tension of the solution‐based soymilk sample ranged from 0.0451 to 0.0466 N/m for 0 to 40% sucrose and that of the solution‐based SDS sample was from 0.0313 to 0.0341 N/m for 40 to 60% sucrose. The viscosity of both the SDS solution and the soymilk solution was almost constant with the rotation speed and showed Newtonian fluid properties. The calculated viscosity of soymilk solutions ranged from 0.0079 to 0.0377 Pa·s for 0 to 40% sucrose. SDS solution viscosity ranged from 0.0052 to 0.0427 Pa·s for 40 to 60% sucrose. The density of soymilk solutions ranged from 1.115 to 1.404 kg/m^3^ for 0 to 40% sucrose and that of SDS solutions ranged from 1.115 to 1.221 kg/m^3^ for 40 to 60% sucrose.

**Figure 3:**
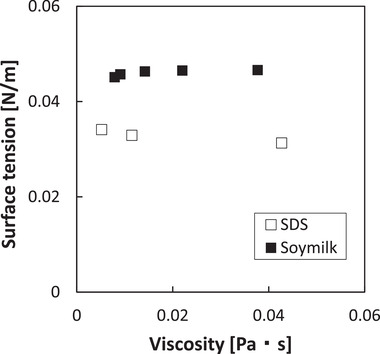
Relationship between viscosity and surface tension of solution.

**Figure 4:**
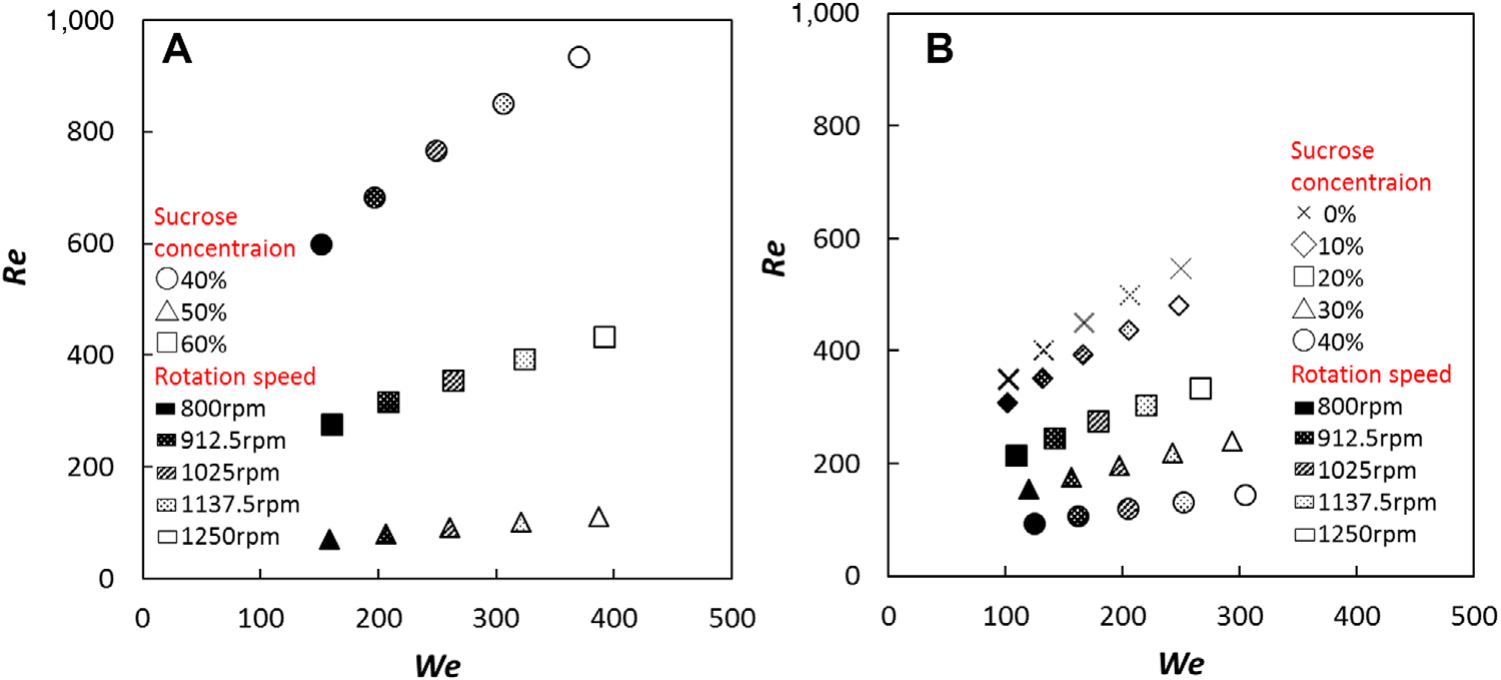
Relationship between *We* and *Re* in SDS solution (A) and in soymilk solution (B).

Although the surface tension and viscosity of each solution were changed with the concentration of sucrose, the correlation between the two was low and not necessarily proportional (Figure 3). Therefore, a comprehensive evaluation using dimensionless numbers was attempted. The Weber number (*We*), Reynolds number (*Re*), and Froude number (*Fr*) were chosen as dimensionless numbers, explaining the liquid behavior. *We*, *Re*, and *Fr* were defined as follows:

(5) $$We\;=\frac{u^{2}d\rho_{L}}{\gamma_{L}}\;$$

(6) $$Re\;=\frac{ud\rho_{L}}{\eta_{L}}\;$$

(7) $$Fr\;=\frac{u^{2}}{gd}\;$$

where *u* is the whipping speed, *ρ_L_* is the liquid density, *d* is the whipper size, *η_L_* is the liquid viscosity, and *γ_L_* is the liquid surface tension. Further, *u* was calculated as follows:

$$u\;=d_{w}\times \pi \times N/60\;$$

where *d_w_* is rotation diameter of whipper (*d_w_* = 37.92 × 10^−3^ m) and *N* is rotation speed per minute. In this study, *d* was constant (*d* = 1.5 × 10^−3^m), but *u*, *ρ_L_*, *η_L_*, *and γ_L_* were variable. Thus, these dimensionless numbers were calculated using five different rotational speeds, three types of surface tension, viscosity, and densities of the SDS solution, and five types of these parameters for the soymilk solution. Therefore, *We* increased against surface tension, *Re* increased against viscosity, and *Fr* increased in proportion to the rotation speed. The relationship between *We* and *Re* is shown in Figure 4. It was shown that *We* greatly changed depending on the rotation speed and that there were solution systems where *Re* also changed greatly with the shift and some where it did not. In all solutions, *Fr* had almost the same proportional relationship regardless of the concentration with *We*, and the relationship with *Re* varied depending on the concentration of the solution. From these results, it was found that *Re* could be changed more than *We* and *Fr* by changing the rotation speed, and there were solution viscosities for which behavior changed according to the rotation speed and some that did not change much.

### 3.3 Whipping behavior

For two‐phase foam, bubble size supposedly depends mainly on four kinds of force, specifically surface tension, viscous force, inertial force, and gravity. Thus, the normalized mean bubble diameter should be described by a function of *We*, *Re*, and *Fr* (Yano, 1987, 1988).

(8) $$\;\frac{d_{bm}}{d}=\;A{\left(We\right)}^{k}{\left(Re\right)}^{l}{\left(Fr\right)}^{m}$$

In this study, rice flour batter was regarded as a three‐phase dispersion system in which particles dispersed into a foam, and it is necessary to consider the information about the solid phase. Therefore, *Bo* and *d_c_cosθ/d_p_* were noted for the analysis of whipping behavior. *Bo* and *We* reflected surface tension, and *d_c_cosθ/d_p_* accounted for wettability of the particle surface, so that the whipping behavior was dependent on the interfacial effect of the solid and liquid phases. Thus, the estimation index *d_bm_/d* including the effect of solid and liquid phases was constructed as follows to understand the whipping behavior based on the coefficient *A* and the power values *k*, *l*, *m*, *n*, and o.

(9) $$\;\frac{d_{bm}}{d}=\;A{\left(We\right)}^{k}{\left(Re\right)}^{l}{\left(Fr\right)}^{m}{\left(Bo\right)}^{n}{\left(\frac{d_{c}cos\theta }{d_{p}}\right)}^{o}$$

**Figure 5:**
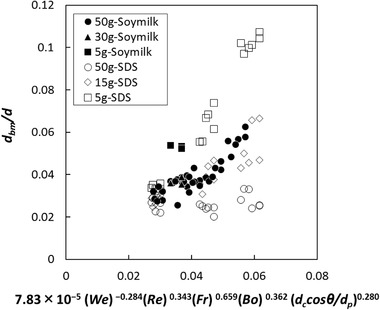
Comparison between experimental data and regression equation.

**Figure 6:**
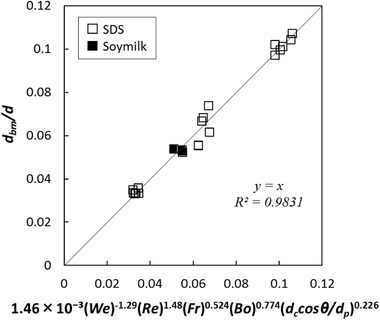
Comparison between experimental data and regression equation when adding 5 g of rice flour.

**Figure 7:**
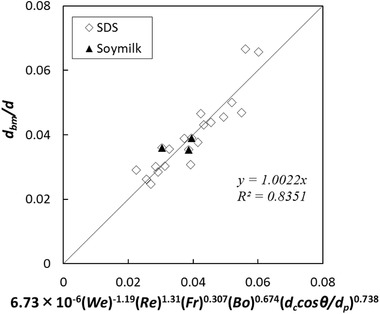
Comparison between experimental data and regression equation when adding 15 to 30 g of rice flour.

**Figure 8:**
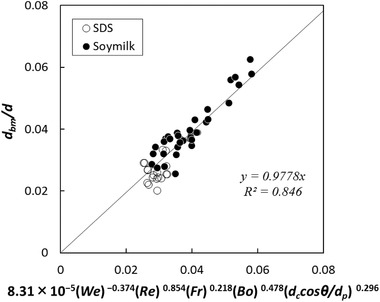
Comparison between experimental data and regression equation when adding 50 g of rice flour.

The coefficient and the power values were calculated for various batters with Eq. 9 based on nonlinear least‐squares curve fitting with Microsoft Excel Solver. The relationship between the normalized mean bubble size and the estimation index is shown in Figure 5. There was no linear correlation common to all samples, and the plot appeared to vary depending on the amount of rice flour. Specifically, the regression equations should be constructed according to the change in the three‐phase dispersion state. Therefore, the calculation was performed by dividing the amount of rice flour addition into a small amount of 5 g, a medium amount of 15 to 30 g, and a large amount of 50 g. A strong linear correlation was observed in all cases (Figures 6, 7, 8). Thus, this analysis successfully demonstrated that bubble size could be estimated by dimensionless parameters. Moreover, the coefficient *A* and the power values *k*, *l*, *m*, *n*, and *o* were compared to understand how the effect of the factors changes with different amounts of rice flour. When the amount of rice flour addition was 5 g, parameters *k* and *l* of *We* and *Re* were high in magnitude (*k* = −1.29, *l* = 1.48). It was shown that the effect of surface tension and viscosity was strong. When the surface tension is low and the viscosity is high, foam with a small bubble diameter can be obtained. For the addition of 50 g, the contribution of solids relatively increased since the values of *k* and *l* became small, the value of *n* decreased, and the value of *o* increased. For the addition of 15 to 30 g, the value became an intermediate value between 5 and 50 g. Previous studies have reported that foam with small diameters are obtained with conditions of high viscosity and low surface tension in two‐phase foam (De Preval, Fabrice, Gilles, Gérard, & Samir, 2014; Mary et al., 2013). For the small addition, the batter might behave like a two‐phase dispersion. With increasing addition, fine particles of rice flour formed membrane‐like structures at the surface of the bubble through their aggregation from dispersion. Then, the effect of solid properties was stronger with high‐density fine particles, and consequently, the contribution of the wettability of rice flour was higher for the bubble size. As Dickinson (2010) reported, particles form membranes at the gas‐liquid interface, and bubbles are stabilized for a long time. The higher the density of particles at the interface, the higher the elasticity of the monolayer that is formed, and the barrier effect is enhanced (Dickinson, 2017).

**Figure 9:**
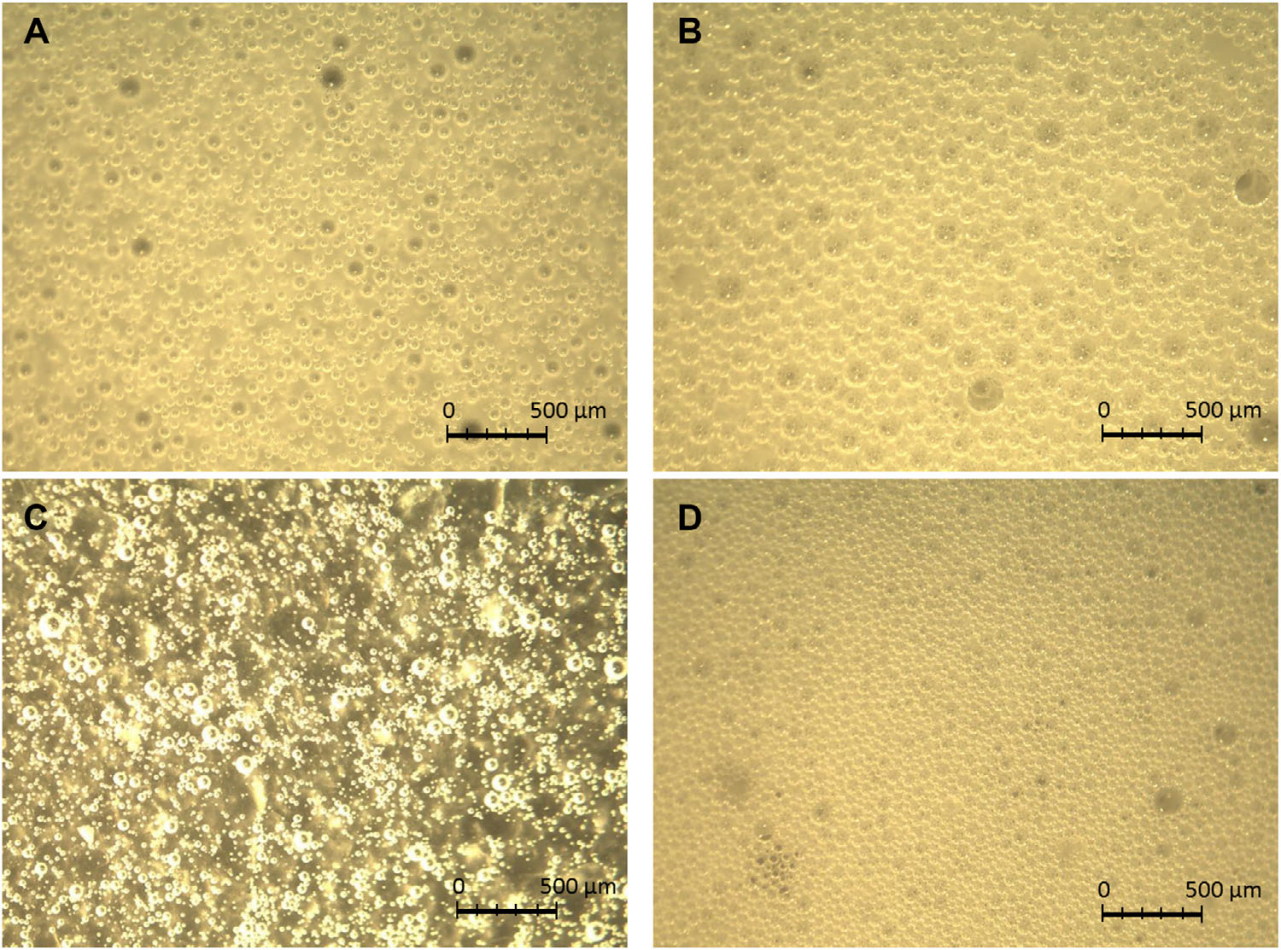
Bubbles in rice flour batter. (A) 50 g of rice flour added in soymilk solution. (B) 5 g of rice flour added in soymilk solution. (C) 50 g of rice flour added in SDS solution. (D) 5 g of rice flour added in SDS solution.

The particle arrangement is determined by the interaction between adjacent particles and the contact angle *θ* with the interface generated on the surface of the particles. The steric hindrance and capillary pressure of the particles are effective factors for holding the particles and the liquid phase in thin film (Dickinson, 2017). When particles are present in excess, multiple layers are formed providing stronger barriers (Dickinson, 2017). It was confirmed that the values of the coefficients *n* and *o* became relatively large with an increase in rice flour. Thus, the bubble diameter was reduced by not only high viscosity and low surface as reported by a previous study (Dickinson, 2017) but also small particle size and low wettability. As Yoza, Okabe, and Shima (2008) noted, the result suggested that rice flour with fine particle‐maintained starch granules can be developed by wet milling. In addition, as Li, Li, Sun, and Yang (2013) reported, rice starch has high emulsifying potential so that the rice flour is very effective for the stabilization of bubbles, especially small bubbles. Moreover, previous studies have reported that starch damage is correlated with wettability (Greer & Stewart, 1959; Matsuki et al., 2015) and that the surface tension of batter decreases with low starch damage (Yano et al., 2017). Therefore, it is expected that small bubbles are retained by suppressing the coalescence of foam. In this study, the milling process was changed to produce rice flours with different powder properties such as particle size, surface wettability, and structure of the starch. Namely, the milling process does not affect the mean bubble size, but these powder properties affect mean bubble sizes in cake batter with a solid‐gas‐liquid system. *Bo* decreased despite the addition of rice flour as shown by index *n*. The result suggested that the effect of *Bo* was weakened by interactions between particles because strong membranes due to rice flour are formed at the surface with an increase in addition. Microscopic images of each solution are shown in Figure 9. The bubble size of soymilk solution and SDS solution was smaller with increasing amounts of rice flour. In addition, the contact among the adjacent bubbles of high‐concentration rice flour (as shown Figure 9A and 9C) was less than that of low‐concentration rice flour (Figure 9B and 9D). Thus, it is very likely that the addition of rice flour is a constraint factor to stabilize the small bubble.

The properties of rice flour are greatly affected by the milling process. In particular, the water absorption varies by each product so that in practical situations, the quality of rice flour‐based products is not constant. Thus, the optimum addition when making the batter should be adjusted depending on the rice flour. To inclusively suggest a control method, it is necessary to develop the estimation model including the effect of powder properties of rice flour in a gas‐liquid solid system. Therefore, the whipping properties of batter were recalculated by Eq. 10 as follows. To correspond to the solid content ratio, the explanatory valuable for the volume fraction of rice flour (*Φ_r_*) was added in Eq. 9.

(10) $$\;\frac{d_{bm}}{d}=\;A{\left(We\right)}^{k}{\left(Re\right)}^{l}{\left(Fr\right)}^{m}{\left(Bo\right)}^{n}{\left(\frac{d_{c}cos\theta }{d_{p}}\right)}^{o}{\left(\Phi_{r}\right)}^{p}$$

the volume fraction of each rice flour (*Φ_r_*) was obtained as follows:

(11) $$\;\Phi_{r}=\frac{m_{r}/\rho_{p}}{\left(m_{r}/\rho_{p}\right)+\left(m_{L}/\rho_{L}\right)}\;$$

where *m_r_* is the mass of rice flour [kg], *ρ_p_* is the density of the rice flour particle [kg/m^3^], *m_L_* is the mass of the liquid [kg], and *ρ_L_* is the density of the liquid [kg/m^3^]. The liquid corresponds to soy milk and SDS, but was calculated by approximating with pure water (i.e., *ρ_L_* was 997 kg/m^3^). The coefficient and the power values were calculated in the same manner as Eq. 9.

**Figure 10:**
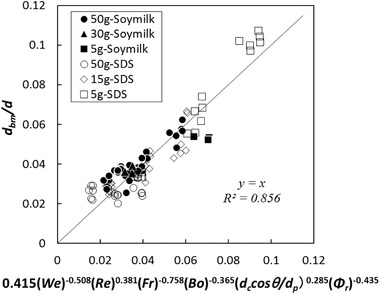
Comparison between experimental data and regression equation.

The relationship between the normalized mean bubble size and the estimation index is shown in Figure 10. A linear correlation was confirmed for all samples when subjected to the addition of different amounts of rice flour. As described previously herein, the interfacial state in the batter greatly differs depending on the amount of rice flour, and it contributes not only to the stability of the bubbles but also their size. Therefore, adjusting the model by the volume fraction of rice flour was possible to predict the bubble diameter from one common equation even in the case of batter. The power value of the volume fraction was a negative number (*P* = −0.435), thus, it is suggested that the increase in solids contributed to form the smaller bubble.

## 4 CONCLUSIONS

The quest for the optimization of process control methods is essential to develop new rice flour‐based cellular foods in response to food allergies. The aim of this study was to investigate the whipping behavior of rice flour batter as a three‐phase dispersion. Six types of rice flour were prepared by different milling processes. To evaluate the powder properties of rice flour, the particle density, surface tension, and wettability were investigated. A two‐dimensional plot of the relationship between *Bo* and the wettability parameter *d_c_cosθ/d_p_* clearly suggested that differences in the powder properties of rice flour could be evaluated by particle size and wettability. The foam strongly depended on the inertial force, viscous force, and surface tension and was affected by solids in the three‐phase dispersion. The normalized mean bubble diameter reflecting whipping behavior was predicted by *We*, *Re*, Fr, *Bo*, and *d_c_cosθ/d_p_*. Therefore, a regression equation was constructed using these dimensionless numbers, and the effects of factors derived from materials and operating conditions were investigated. No regression equation had a linear relationship with the bubble diameter in all the prepared samples, but empirical regression equations, were obtained according to the amount of rice flour addition. It was predicted that when the amount of added rice flour was small, the effect of liquid properties was large and that high viscosity and low surface tension of the liquid phase made a smaller bubble diameter. Further, it was considered that the effect of the solid increased as the amount of added rice flour increased and that the small particle size and low wettability were the conditions required for reducing bubbles. In the solid‐gas‐liquid three‐phase dispersion system, it is possible to analyze the whipping characteristics by the dimensionless parameters *We*, *Re*, *Fr*, *Bo*, and *d_c_cosθ/d_p_* in the equation according to the amount of solid addition. The dimensionless mean diameter (*d_bm_/d*) was expressed by a dimensionless equation. These results successfully demonstrated the potential to evaluate the whipping process by dimensionless parameters. To improve the prediction accuracy by the empirical equation, it is very important to obtain information about the bubble size in batter from versatile flours that have different powder properties or materials and conditions. Moreover, the contribution of the breaking process to the sizes of bubbles formed by the whipping process should be clarified. The state of the bubble nucleus in a food material is important to evaluate the initial swelling process during baking. Thus, understanding the effect of bubble properties, particularly bubble diameter in the batter and the growth of bubbles in the swelling process, might lead to the optimization of processing for rice flour‐based foods and a new cooking method.

## AUTHOR CONTRIBUTIONS

T. Fujii designed the study and interpreted the results. C. Ichikawa and D. Ishikawa collected test data and drafted the manuscript. J. Yang collected test data.

## CONFLICT OF INTEREST

The authors declare no conflict of interest.

## Nomenclature

*A*: coefficient of demensionless equation [‐]
*Bo*: Bond number [‐]
*Ccosθ*: wetting parameter [m³]
*d*: whipper size [m]
*d_bm_*: mean bubble size [m]
*d_c_cosθ*: wettability parameter [m]
*d_c_cosθ/d_p_*: dimensionless parameter of wettability [‐]
*d_p_*: mean particle size of rice flour [m]
*d_w_*: rotation diameter of whipper [m]
*Fr*: Froude number [‐]
*g*: gravity [m/s²]
*k*: power value of *We* [‐]
*l*: power value of *Re* [‐]
*m*: power value of *Fr* [‐]
*m_r_*: mass of rice flour [g]
*m_L_*: mass of liquid in batter [g]
*N*: rotation speed per minute [min^−1^]
*n*: power value of *Bo* [‐]
*o*: power value of *d_c_cosθ/d_p_* [‐]
p: power value of $\Phi_{r}$ [‐]
*r*: capillary radius [m]
*Re*: Reynolds number [‐]
*S*: cross‐sectional area of the powder bed [m²]
*t*: hydration time [s]
*u*: whipping speed [m/s]
*V*: hydration volume [m³]
*We*: Weber number [‐]
*γ_L_*: liquid surface tension [N/m]
*γ_w_*: water surface tension [N/m]
*ε*: porosity [%]
*η_L_*: liquid viscosity [kg/m·s]
*η_w_*: water viscosity [kg/m·s]
*θ*: contact angle [°]
*ρ_p_*: particle density [kg/m³]
*ρ_L_*: liquid density [kg/m³]
$\Phi_{r}$: volume fraction of rice flour in liquid phase [‐]
